# Development of Physical Performance Tasks during Rapid Growth in Brazilian Children: The Cariri Healthy Growth Study

**DOI:** 10.3390/ijerph16245029

**Published:** 2019-12-10

**Authors:** Simonete Silva, Hudday Mendes, Duarte Freitas, António Prista, Go Tani, Peter T. Katzmarzyk, Adam D. G. Baxter-Jones, Alcibíades Bustamante Valdivia, José Maia

**Affiliations:** 1 Department of Physical Education, University Regional of Cariri, Ceará 63105-000, Brazil; simonete.silva@urca.br (S.S.); hudday.mendes@urca.br (H.M.); 2 Department of Physical Education and Sports, University of Madeira, 9000-082 Funchal, Portugal; dfreitas@staff.uma.pt; 3 CIFI2D, Faculty of Sport, University of Porto, 4200-450 Porto, Portugal; 4 Research Group for Physical Activity and Health, FEFD-CIDAF, Pedagogical University, Maputo 1106, Mozambique; aprista1@gmail.com; 5 School of Physical Education and Sports, University of São Paulo, São Paulo 05508-030, Brazil; gotani@usp.br; 6 Pennington Biomedical Research Center, Louisiana State University, Baton Rouge, LA 70808, USA; peter.katzmarzyk@pbrc.edu; 7 College of Kinesiology, University of Saskatchewan, Saskatoon, SK S7N 0W6, Canada; baxter.jones@usask.ca; 8 Faculty of Physical Culture and Sports, Universidade Nacional de Educación Enrique Guzman y Valle, 60637 La Cantuta, Lurigancho-Chosica 15472, Peru; huanta2609@yahoo.es

**Keywords:** children and adolescents, peak height velocity, peak physical performance, growth rate

## Abstract

Growth and physical performance scores were studied around three years of attainment of peak height velocity (PHV). We aimed to estimate the age at peak velocity, or at peak rate, in physical performance tasks, and sex-differences when aligned by biological age. A total of 131 boys and 123 girls, 8 to 14 years of age were recruited from the Cariri region of Brazil. A mixed longitudinal design was used with four overlapping age cohorts: 8, 10, 12, and 14 years, followed for three years, with measurements performed at 6 month intervals. Height, 12 min run (12mR), handgrip strength (HG), standing long jump (SLJ), and shuttle run (SR) velocities were estimated using a non-smooth mathematical procedure. Age at PHV was 13.4 ± 1.6 years in boys compared with 12.2 ± 2.3 years in girls. Maximal velocity in SLJ was attained 6 and 3 months prior to PHV in boys and girls, respectively. For HG, peaks were attained 9 months after PHV in boys and 15 months after PHV in girls. Maximal velocity in 12mR was attained 6 months before PHV in boys and at PHV in girls, whereas a peak in SR occurred 12 months after PHV in both sexes. In conclusion, dynamic changes in physical performance relative to PHV appear similar in both sexes, although sex differences were evident in some motor tests.

## 1. Introduction

It has been shown that maturity status is associated with physical performance task development, particularly in relation to the timing of the adolescent growth spurt in height [1]. This is important, as moderate-to-high levels of physical performance during periods of rapid growth in adolescence are associated with health markers [2]. However, interpretation of these associations may be problematic given the inter-individual differences in physical performance measures within and between populations. For example, in Peruvian [3], Brazilian [4] and Portuguese [5] children and youth, there are systematic differences in physical performance centiles in subjects of the same chronological age. This suggests that studies of development in physical performance need to incorporate the known variability associated with normal biological maturation [1]. Thus, alignment of individuals on a marker of biological age, such as biological age calculated from peak height velocity (PHV), rather than chronological age, is imperative, as it is well known that there is considerable variability in PHV timing, intensity, and duration between and within sexes [6].

Although the adolescent growth spurt in height has been studied extensively, less is known with regards to the variability of the timing, intensity, and duration of peak velocities in physical performance tasks. It has been reported that boys at a given chronological age who are advanced in maturation demonstrate better physical performance, in absolute terms, than their later-maturing peers. Interestingly, late matures tend to catch up to their early maturing peers and can achieve better performance outcomes in early adulthood [7]. 

Age of attainment of PHV (age-at-PHV) is a maturational milestone that is common to both boys and girls. Several studies have used age-at-PHV to investigate age- and maturity-related changes in physical growth, body composition [8], muscular strength [9], and maximal aerobic power [10,11]. However, very few studies have aligned physical performance test batteries on indicators of biological age. Previously, Belgian boys [1], Spanish boys and girls [12], and Belgian soccer players [13] have shown inconsistent peak velocities in physical performances. Studies among girls are relatively scarce, but in an early study by Espenschade [14], it was found that performance in several physical performance tests remained quite stable before, at, and after menarche. Further, Kemper and Verschuur [10] suggested that peak growth in static arm strength in girls peaked one year following PHV, although they did not align their data on age-at-PHV in terms of velocities (kg·year^−1^). In contrast, Faust [15] stated that “the timing of maximum velocity in strength for girls was highly variable and did not correspond closely with the pubertal period of height growth” (p.59).

This study aims to (1) estimate the age at peak velocity in physical performance tasks aligned by biological age and (2) to determine whether any sex-related differences exist.

## 2. Materials and Methods

### 2.1. Design and Participants

Subjects were recruited from the Cariri Healthy Growth Study, which has been described in detail elsewhere [4]. Briefly, Cariri is a region in the state of Ceará, Brazil and comprises nine cities. This region is characterized by a low human development index (HDI) when compared with the south, southeast, and mid-west regions of Brazil [16]. Geo-climatic, socioeconomic, and cultural characteristics are different from those of other Brazilian regions [17]. The population of Cariri is quite heterogeneous due to the high rate of immigration from other northeastern Brazilian states, mostly for religious reasons. Since we did not have genetic data to characterize the composition of our sample, we precluded ourselves from classifying the children and adolescents into groups simply based on their skin color.

This study used a mixed longitudinal design, with four overlapping age cohorts: cohort 1 starting at 8 years, cohort 2 starting at 10 years, cohort 3 starting at 12 years, and cohort 4 starting at 14 years at baseline. Subjects were then followed for three consecutive years, with measurements taken at six-month intervals from 2006 through 2009. The sample comprises 498 subjects (237 girls and 261 boys), recruited from three cities (Barbalha, Crato, Juazeiro do Norte), and from urban areas. These cities are similar to others in the region. For example, the mean HDI for all cities in the region is 0.642, varying from 0.578 (Caririaçu) to 0.713 (Crato). The HDI in the three cities are Barbalha, HDI = 0.683, Juazeiro do Norte, HDI = 0.694, Crato, HDI = 0.713; all are located in the metropolitan area of Cariri and each city has the following geographical location: Juazeiro do Norte (Latitude: 07°12′47″ S, Longitude: 39°18′55″ W), Crato (Latitude: 7°13′46″ S, Longitude: 39°24′32″ W), and Barbalha (Latitude: 7°18′20″ S, Longitude: 39°18′9″ W).

Only subjects from cohorts 2, 3, and 4 on whom age-at-PHV was estimated and who had complete data on physical performance tasks were considered in the analysis. A total of 131 boys and 123 girls fulfilled these criteria. The study was conducted according to the guidelines of the Brazilian norms (Res. 466/2012-CNS). All procedures were approved by the Ethics Research Committee of the Medical School of Juazeiro do Norte (CEP-FMJ 01/07) and the directors of the schools. Informed consent was obtained from parents, and assent to participate was obtained from the children.

### 2.2. Measurements and Tests

#### 2.2.1. Anthropometry

Height was measured, using standardized techniques [18], with a portable stadiometer (CARDIOMED^®^Welmy Model 220, Cardiomed, Curitiba, Brazil) to the nearest 0.1 cm, with the head positioned in the Frankfurt plane.

#### 2.2.2. Physical Performance

Physical performance was assessed through a measure of handgrip (static strength using Takei Hand Grip Dynamometer^®^, Takei Scientific Instruments Co., Ltd., Nigata, Japan), standing long jump (explosive power), shuttle run, 10 × 5 m (running speed and agility), and 12 min run (cardiorespiratory endurance). The first three tests were from EUROFIT [19], and the 12 min run from The American Alliance for Health, Physical Education and Recreation [20].

### 2.3. Data Quality Control

Data quality control followed a three-step process. First, a pilot study was conducted to assess standardization of all measurements. Second, intra-individual test–retest reliability was carried out on three to five children, randomly selected every day, and ANOVA-based intraclass correlations (R) were computed. The intra-observer technical error of measurement was 0.5 cm for height; whereas for the physical performance tests, R ranged from 0.85 (95% CI = 0.79–0.89) for shuttle run to 0.97 (95% CI = 0.95–0.98) for standing long jump. Third, data entry errors and inconsistent records were identified and corrected. Data cleaning was performed in IBM SPSS, version 21.0 (IBM Corp., 2012, SPSS Inc., Chicago, IL, USA).

### 2.4. Statistical Procedures

Individual velocity data for height and physical performance tests were fitted with a modified non-smoothed polynomial method. This method was firstly presented by Beunen, Malina, Van’t Hof, Simons, Ostyn, Renson and Van Gerven [1] with adolescent Belgian boys and subsequently used by Yague and De La Fuente [12] in Spanish boys and girls, and by [13] in Belgian male soccer players. Following this approach, a mathematical generalization of the method was developed. Although measurements were taken every six months, the method permits the estimation of individual velocities every three months. Mean velocity curves (also called mean constant curves) were developed and defined in terms of time, that is, months before and after age-at-PHV. Growth velocities were estimated using software developed by a mathematician and software programmer from the University of Porto. Graphical data were computed using a cubic spline procedure (GraphPad Prism version 3.00 for Windows, GraphPad Software, San Diego, CA, USA). A cubic spline employs interpolating cubic polynomials, which use information from neighboring points to obtain a degree of global smoothness. The cubic spline procedure was chosen over other curve-fitting protocols because it maintains the integrity of the data without transforming or modifying the underlying growth characteristics.

## 3. Results

Mean increments at PHV in boys and girls were slightly different (see Table 1). In boys, height velocity increased from 2.69 cm·year^−1^ (18 months before PHV) to 8.49 cm·year^−1^ at PHV (age-at-PHV = 13.4 ± 1.6 year). For girls, height velocity increased from 2.22 cm·year^−1^ at 18 months before PHV to 7.26 cm·year^−1^ at PHV (age-at-PHV = 12.2 ± 2.3 year).

**Figure 1:**
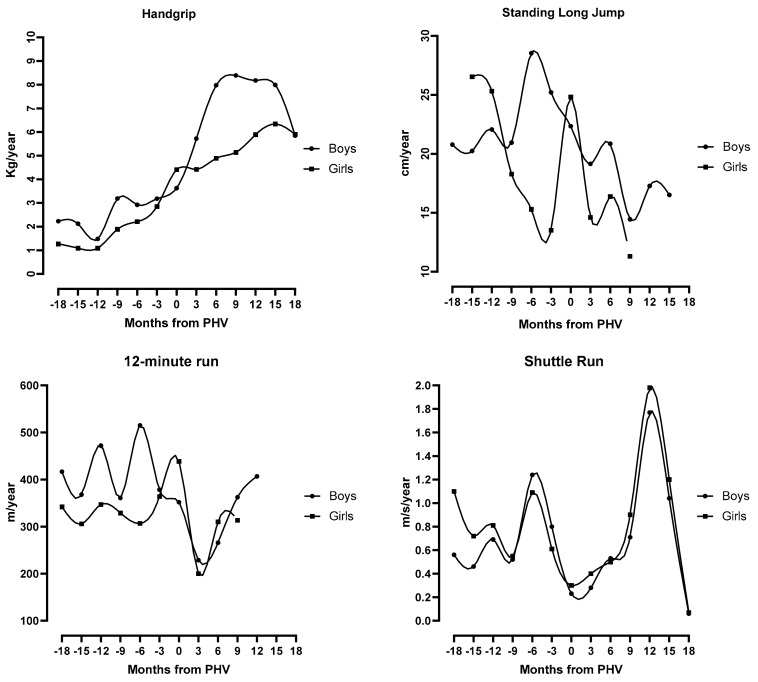
Boys and girls physical performance mean constant velocity curves for hand grip, standing long jump, 12 min run and Shuttle Run.

Table 2 displays the results for the mean constant curves, and Figure 1 presents the smoothed cubic splines for the physical performance data. For handgrip (static strength), velocity increased gradually in boys and girls. In boys, the curve displayed acceleration from 2.23 kg·year^−1^ (18 months before PHV) to 8.39 and 8.18 kg·year^−1^ (9 to 12 months after PHV) and declines thereafter. In girls, there was an increase from 1.27 kg·year^−1^ (18 months prior to PHV) to 6.34 kg·year^−1^ (15 months after PHV). Boys’ standing long jump (explosive power) reached its peak 6 months before PHV (28.54 cm·year^−1^), whereas girls reached three apparent peaks: at 15 and 12 months before PHV (26.54 and 25.32 cm·year^−1^, respectively) as well as coincident with PHV (24.83 cm·year^−1^). The estimated velocity curves for 12 min run (cardiorespiratory endurance) showed a fluctuation in mean values when aligned by PHV. Boys reached their peak 6 months before PHV (515.07 m·year^−1^); then the velocity declines, but 12 months after PHV the velocity increases again to 406.72 m·year^−1^. Girls reached their peak coinciding with PHV (438.46 m·year^−1^), and then declined thereafter. For shuttle run (running speed and agility), boys and girls attained their peak 12 months after their PHV. Maximal velocities were 1.77 m·s^−1^·year^−1^ and 1.98 m·s^−1^·year^−1^ for boys and girls, respectively.

Table 3 summarizes the data on the timing of attained PHV and peak velocities in physical performance tasks. Annual increments in boys were higher than in girls in height, and timing of the peaks were different and statistically significant (*p* < 0.05) for height, handgrip, standing long jump, and 12 min run.

## 4. Discussion

In this study, we have shown distinct patterns in peaks in physical performance occurrence relative to PHV in a sample of Brazilian children with a low HDI. This information is unique given the lack of available longitudinal studies of biological age-related trends in physical performance, especially in developing nations. Further, the most recent data on nonathletic children on this issue dates back to 1983–1988 [21] from research conducted in Menorca Island, Spain, that was later used by Yague and De La Fuente [12].

It is well known that growth and maturation can be influenced by HDI status [22]. Further, recent data about growth spurt is apparently not available, and so we relied on results dated from 20 to 50 years ago. In the current sample of low HDI children, average age-at-PHV for boys (13.4 year) was relatively similar to that of Spaniards (13.0 ± 0.63 year) [12] and Canadians (13.4 ± 1.0 year) [23], respectively, but relatively earlier than that of Swiss (13.9 ± 0.8 year) [24], Swedish (14.2 ± 0.5 year) [25] and British (14.1 ± 0.13 year) boys [26]. The estimated age-at-PHV of Brazilian girls was 12.2 year and parallels those reported in Swiss (12.2 ± 1.0 year) [24], Spaniards (12.4 ± 0.57 year) [12], and in British girls (12.1 y ± 0.14) [26]. In contrast, girls showed minor differences in age-at-PHV relative to Swedish [25] and Canadian girls [23], 12.0 ± 0.40 year and 11.8 ± 0.90 year, respectively. It is noteworthy that Brazilian girls’ age-at-PHV coincides with that of Europeans and Canadians, and Brazilian boys reached their PHV at similar and earlier ages than several European samples. Further, the PHV of Brazilian boys (8.39 cm·year^−1^) and girls (7.26 cm·year^−1^) is within the normal range reported by Malina, et al. [27], that is, 8–10 cm·year^−1^ in boys and 7–9 cm·year^−1^ in girls. In making these comparisons, it is important to be aware of the temporal gap between studies, the positive trends in age-at-PHV [28], differences in socioeconomic status among the children, study duration, and the curve-fitting models used for analysis, all of which can affect the estimates of parameters of the growth spurt. It is also possible that the time lag of the present study, the region’s HDI, the sample size, as well as the mathematical model used may be responsible for the small differences in boys and girls mean ages at PHV. Since we do not have comparable data from Brazilian youth or other South American samples, we cannot know if these mean ages-at-PHV in Cariri boys and girls can be classified as early and/or late maturing. 

Aligning different expressions of physical performance relative to age-at-PHV is problematic as valid data varies substantially from the original sample size and is different whatever the considered physical performance marker one uses [11,12,29]. In the present study, we also faced the same problem and acknowledge that this could affect the interpretation of our physical performance tests. Boys’ and girls’ hand grip strength increased gradually until their peak, which occurred at 9–12 months after age-at-PHV in boys (8.39 to 8.18 kg·year^−1^) and 12 months after PHV in girls (6.34 kg·year^−1^). Using maximum voluntary contractions (MVC) in elbow and knee extensor muscles assessed longitudinally from 8–17 years of age, Round, et al. [30] aligned British boys’ and girls’ distance curves by age-at-PHV and showed clear differences between them, mostly evident after age-at-PHV, although they did not investigate the existence of strength spurts. Additionally, Beunen, Malina, Van’t Hof, Simons, Ostyn, Renson and Van Gerven [1], using arm pull strength as a marker of static strength of Belgium boys, revealed that its spurt, when aligned by age-at-PHV or age at peak weight velocity (PWV), occurred 6 months after PHV or after PWV. Also, Carron and Bailey [9] reported that various measures of Canadian boys’ static composite strength peaks 12 months after age-at-PHV and overlaps age at peak weight velocity (age-at-PWV), indicating that the increases in muscle mass likely play an important role in this peak, added to this, increases in plasma testosterone could also explain the differences [30]. As a final remark, Iuliano-Burns, Mirwald and Bailey [23] clearly showed that function follows form, where bone increases in length precede muscle mass increases and these jointly affect force production.

Boys’ peak spurt in standing long jump (28.54 cm·year^−1^) occurred 6 months before age-at-PHV, whereas in girls, a possible plateau occurred from 15 to 12 months prior to PHV (26.54 to 25.32 cm·year^−1^) to being coincident with PHV (24.83 cm·year^−1^). In Spanish boys, there was a plateau at 21–22 cm·year^−1^ from age-at-PHV until 12 months afterwards, whereas in girls, the peak spurt was evident 4 months after age-at-PHV (12 cm·year^−1^) [12]. In contrast, [31] found a peak (15.24 cm·year^−1^) in Canadian boys coincident with their age-at-PHV. Using a different marker of explosive strength (vertical jump), Beunen, Malina, Van’t Hof, Simons, Ostyn, Renson and Van Gerven [1] showed, in Belgium boys, a peak 6 months after PHV and PWV. This discrepancy in timing and intensity of peaks may be due to sample specificities as well as measurement frequency. Different levels of motor skill and coordination, and distinct intensities and timings of growth velocities of the leg length, as well as differences in muscle mass may be at work to explain these results.

For the 12 min run, girls obtained their peak spurt (438.46 m·year^−1^) coincident with their age-at-PHV, whilst boys reached it 6 months before their age-at-PHV (515.07 m·year^−1^). It is difficult to compare the magnitude of these results with other samples since no similar data are available. However, Yague and De La Fuente [12] used a 6 min run test and found rather different results. In boys, from a negative velocity at 12 months before age-at-PHV (−12 m·year^−1^), they reached their peak about 8 months after age-at-PHV (140 m·year^−1^), and the velocity declined drastically (30 m·year^−1^) thereafter. Although in Brazilian boys the velocity pattern in slightly different, no negative values were found; besides, after the peak spurt, a decline is evident till 6 months after age-at-PHV, and then a rise can be seen at 12 months. In Spanish girls, the highest velocity occurs 8 months before age-at-PHV (120 m·year^−1^), and then declines drastically till zero at PHV. Brazilian girls show a peak spurt (438.46 m·year^−1^) coincident with age-at-PHV. In Belgium twins of both sexes, [11] found a peak VO_2_ (L·min^−1^·year^−1^) in aerobic power coincident with their age-at-PHV. In Canadian active and inactive boys, Mirwald, et al. [32] identified a peak slightly after age-at-PHV. In both studies, Preece-Baines I was used to model the growth curves. 

For the shuttle run, boys and girls had a performance peak at 12 months after age-at-PHV (1.77 m·s^−1^·year^−1^ in boys and 1.98 m·s^−1^·year^−1^ in girls), whereas Spanish boys and girls reached their peak 8 months before age-at-PHV and 4 months after age-at-PHV (0.68 s·year^−1^ and 0.48 s·year^−1^, respectively). Belgian boys had an apparent peak (1.6 s·year^−1^) approximately 2 years before age-at-PHV [1]. These differences in peaks may be explained by the specificities of each study. Of interest, the Belgian and Spanish data were expressed differently, that is, changes in time (s·year^−1^). We contend that expressing it as m·s^−1^ best describes eventual spurts in velocity. Inter-individual differences in motor coordination may also explain these distinct peaks, although there is no longitudinal study available in this age span. Starosta and Hirtz [33] attempted to identify sensitive periods in motor coordination and suggested that they possibly occur before PHV. Yet, most of their analyses were based on averages across chronological age of cross-sectional data as well as very small longitudinal data sets and did not provide velocities for any of the coordination tests.

This study is not without limitations. First, it is acknowledged that the sample size is relatively small. Second, this study used a mixed longitudinal design with only three data waves per cohort, which limited the identification of height and physical performance spurts in some children. However, measurements were taken every six months, which allows greater precision in estimating velocities. Yet, Cole [34] concluded that “annual measurements are sufficient to estimate age at peak height velocity to high precision”. Third, the inevitable loss of subjects over time is a common weakness of this research design. Nonetheless, no selective drop-out was identified because no significant differences were found between those who remained in the study as compared with those who dropped out. Fourth, although the 12 min run or the peak of aerobic power mark different aspects of cardiorespiratory endurance, the 12 min run shows acceptable concurrent validity, as reported in a systematic review/meta-analysis by Mayorga-Vega, et al. [35]. Fifth, the sample is not representative of all Brazilian children and adolescents, and care must be taken when trying to generalize these findings.

## 5. Conclusions

In summary, growth spurts in static strength, explosive power, running speed and agility, and cardiorespiratory endurance did not express themselves at the same time relative to age-at-PHV, although they were in line with what would be expected from the known changes in growth and body composition. In boys, a pattern was found in static strength and running speed and agility (9–12 months after age-at-PHV), as well as in explosive power and cardiorespiratory endurance (6 months before age-at-PHV). In girls, the pattern was more evident in static strength, explosive power, and cardiorespiratory fitness (12–15 months after age-at-PHV). Variation in physical performance spurts and sex differences may help physical education teachers and sports coaches to understand the motor development of our children and adolescents and plan for more efficient intervention programs in the school setting or in the sports fields. The results further emphasize that educators should assess individuals’ physical performance development relative to their biological rather than their chronological age.

## Figures and Tables

**Table 1:** Mean height velocities aligned by months from peak height velocity (PHV) in boys and girls.

		Months from PHV												
Variable		−18	−15	−12	−9	−6	−3	0	3	6	9	12	15	18
		**Boys**												
Height (cm·year^−1^)	mean	2.69	3.69	3.42	4.11	5.22	7.26	8.49	7.3	5.34	5.32	5.20	3.49	2.64
	sd	2.68	1.97	2.28	2.19	2.61	2.5	4.02	3.07	3.27	2.7	2.65	1.97	2.64
	*n*	9	19	33	57	84	95	131	79	79	57	42	10	10
		**Girls**												
Height (cm·year^−1^)	mean	2.22	2.98	3.64	4.10	4.66	6.00	7.26	5.73	3.78	3.20	2.87	2.30	2.57
	sd	1.38	1.96	2.71	1.90	2.46	2.95	4.11	2.40	2.32	2.11	2.16	1.50	0.97
	*n*	9	18	32	47	71	85	123	78	77	42	31	2	3

**Table 2:** Physical performance velocities aligned by months from peak height velocity (PHV) in boys and girls.

		Months from PHV												
Variables		−18	−15	−12	−9	−6	−3	0	3	6	9	12	15	18
		**Boys**												
Handgrip (kg·year^−1^)	mean	2.23	2.12	1.48	3.19	2.92	3.18	3.63	5.72	7.98	8.39	8.18	7.99	5.85
	*n*	19	20	45	50	85	90	95	77	65	46	32	24	18
Standing long jump (cm·year^−1^)	mean	20.78	20.24	22.07	20.95	28.54	25.21	22.34	19.15	20.86	14.45	17.28	16.51	*n*/*a*
	*n*	9	16	30	36	57	64	61	38	34	26	15	7	
12 min run (m·year^−1^)	mean	416.73	367.91	472.07	360.96	515.07	378.42	351.81	228.56	265.82	362.34	406.72	*n*/*a*	*n*/*a*
	*n*	7	9	21	26	24	35	29	21	17	11	6		
Shuttle run (m·s^−1^·year^−1^)	mean	0.56	0.46	0.69	0.52	1.24	0.80	0.23	0.28	0.53	0.71	1.77	1.04	0.06
	*n*	9	15	34	37	66	71	65	66	45	53	34	28	19
		**Girls**												
Handgrip (kg·year^−1^)	mean	1.27	1.09	1.09	1.89	2.21	2.85	4.41	4.42	4.89	5.14	5.89	6.34	5.90
	*n*	18	18	44	48	75	78	87	76	62	42	31	18	14
Standing long jump (cm·year^−1^)	mean	n/a	26.54	25.32	18.28	15.29	13.51	24.83	14.61	16.37	11.31	*n*/*a*	*n*/*a*	*n*/*a*
	*n*		10	16	26	24	36	26	22	15	7			
12 min run (m·year^−1^)	mean	341.94	305.63	346.92	328.89	306.73	364.17	438.46	200.15	310.27	313.51	*n*/*a*	*n*/*a*	*n*/*a*
	*n*	4	9	4	23	13	22	19	11	19	7			
Shuttle run (m·s^−1^·year^−1^)	mean	1.10	0.72	0.81	0.55	1.09	0.61	0.30	0.40	0.50	0.90	1.98	1.2	0.07
	*n*	14	17	26	37	45	54	64	60	46	45	34	27	16

Note: Number of subjects (*n*) can vary between tests at successive three-month intervals before and after age-at-PHV. As suggested (Philippaerts et al., 2006), boys and girls whose maximal velocity points were located at the extremes were excluded because it is likely that the real maximal velocity was located before 18 months or after 18 months of age-at-PHV. Peak velocity values are in bold. *n*/*a* = not enough information to estimate this value.

**Table 3:** Timing and peak velocities for physical performance tests in boys and girls.

	Boys		Girls	
Variables	Timing	Peak Velocity (Unit·Year^−1^)	Timing	Peak Velocity (Unit·Year^−1^)
Handgrip	9 to 12 months after PHV	8.39–8.18 kg·year^−1^	15 months after PHV	6.34 kg·year^−1^
Standing long jump	6 months before PHV	28.54 cm·year^−1^	15 to 12 months before PHV, as well as at moment of PHV	26.54 to 25.32 cm·year^−1^; 24.83 cm·year^−1^
12 min run	6 months before PHV	515.07 m·year^−1^	At moment of PHV	438.46 m·year^−1^
Shuttle run	12 months after PHV	1.77 m·s^−1^·year^−1^	12 months after PHV	1.98 m·s^−1^·year^−1^
